# The Role of Cutaneous Microcirculatory Responses in Tissue Injury, Inflammation and Repair at the Foot in Diabetes

**DOI:** 10.3389/fbioe.2021.732753

**Published:** 2021-09-14

**Authors:** Gayathri Victoria Balasubramanian, Nachiappan Chockalingam, Roozbeh Naemi

**Affiliations:** Centre for Biomechanics and Rehabilitation Technologies, Staffordshire University, Stoke-on-Trent, United Kingdom

**Keywords:** diabetic foot, foot ulcer, microvessels, post-occlusive reactive hyperaemia, pressure-induced vasodilation, LDI flare, plantar soft tissues

## Abstract

Diabetic foot syndrome is one of the most costly complications of diabetes. Damage to the soft tissue structure is one of the primary causes of diabetic foot ulcers and most of the current literature focuses on factors such as neuropathy and excessive load. Although the role of blood supply has been reported in the context of macro-circulation, soft tissue damage and its healing in the context of skin microcirculation have not been adequately investigated. Previous research suggested that certain microcirculatory responses protect the skin and their impairment may contribute to increased risk for occlusive and ischemic injuries to the foot. The purpose of this narrative review was to explore and establish the possible link between impairment in skin perfusion and the chain of events that leads to ulceration, considering the interaction with other more established ulceration factors. This review highlights some of the key skin microcirculatory functions in response to various stimuli. The microcirculatory responses observed in the form of altered skin blood flow are divided into three categories based on the type of stimuli including occlusion, pressure and temperature. Studies on the three categories were reviewed including: the microcirculatory response to occlusive ischemia or Post-Occlusive Reactive Hyperaemia (PORH); the microcirculatory response to locally applied pressure such as Pressure-Induced Vasodilation (PIV); and the interplay between microcirculation and skin temperature and the microcirculatory responses to thermal stimuli such as reduced/increased blood flow due to cooling/heating. This review highlights how microcirculatory responses protect the skin and the plantar soft tissues and their plausible dysfunction in people with diabetes. Whilst discussing the link between impairment in skin perfusion as a result of altered microcirculatory response, the review describes the chain of events that leads to ulceration. A thorough understanding of the microcirculatory function and its impaired reactive mechanisms is provided, which allows an understanding of the interaction between functional disturbances of microcirculation and other more established factors for foot ulceration.

## Diabetes Is a Global Health Issue

Diabetes is a common condition which has a considerable impact on the health and economy of nations around the world. There is an annual upsurge in the number of patients being diagnosed with diabetes. The International Diabetes Federation estimates that total global health-care spending on diabetes more than tripled over the period 2003 to 2013 (World Health Organization, 2016). The estimated direct annual cost of diabetes to the world is more than US$ 827 billion and the projected losses in gross domestic product (GDP) for the period 2011 to 2030 is a total of US$ 1.7 trillion worldwide incurred by both the direct and indirect costs (World Health Organization, 2016). This indicates that diabetes imposes a large economic burden on the global health-care system and the wider global economy. As diabetes is a chronic condition, many complications arise as the disease progresses.

## Diabetes Complications and the Role of Microcirculation

Microcirculation is vital for the efficient exchange of gases and nutrients and the removal of the waste products of metabolism. In addition, the cutaneous microcirculation plays an important role in thermoregulation (Flynn and Tooke, 1992). Some of the common complications of diabetes are retinopathy, neuropathy, nephropathy, peripheral vascular diseases, and diabetic foot syndrome. One of the important aspects that resonate with all these complications is microcirculation. Endothelial damage and dysfunction of the microvasculature have been observed in various parts of the body such as the eyes, kidneys and the foot in people with diabetes (Goldenberg et al., 1959; Flynn and Tooke, 1992; Hile and Veves, 2003; Williams et al., 2004; Boulton et al., 2006; Schramm et al., 2006; Körei et al., 2016). Both structural and functional microvascular disturbances (known as microangiopathy or disease to small blood vessels) are commonly observed in people with diabetes as a result of glycation related changes that occur due to the prolonged hyperglycaemic state (Boulton et al., 2006; Singh et al., 2014; Stirban et al., 2014). Besides, glycation related direct changes in the microvessels, both sensory and autonomic neuropathies contribute to the functional changes of the microvasculature (Schramm et al., 2006). As early as in 1983 Parving et al. introduced the “haemodynamic theory” to explain microangiopathy in diabetes (Flynn and Tooke, 1992; Veves et al., 2006; Chao and Cheing, 2009). The theory proposes that the increased microvascular blood flow triggers endothelial injury response, followed by microvascular sclerosis (Flynn and Tooke, 1992; Veves et al., 2006). This, in turn, may lead to functional abnormalities such as impaired maximum hyperaemic response, reduced tissue response to injury or trauma, autoregulation of blood flow and changes to vascular tone (Flynn and Tooke, 1992; Boulton et al., 2006; Veves et al., 2006).

## Diabetic Foot Disease as a Significant Complication and the Role of Microcirculation

In the foot, the adverse complications of diabetes are ulceration and amputation. The annual population-based incidence of diabetic foot ulcers is estimated to be 1.9–2.2% (Levin et al., 2008). Once the skin on the foot is ulcerated, it is susceptible to infections leading to an urgent medical problem (Bakker et al., 2016). It is estimated that only two-thirds of diabetic foot ulcers will eventually heal, but approximately 28% may result in some form of lower extremity amputation (Bakker et al., 2016). Hence, understanding the risks associated with foot ulcer development and its course is crucial. While the role of peripheral vascular disease and neuropathy resulting in diabetic foot ulcers is well-established, more research is needed to understand the contribution of microcirculation (Schaper et al., 2016).

The role of microcirculation in diabetic foot ulcers is a continuing area of research, where there are many theories put forth by several studies on microcirculation and the concept of “small vessel disease” was proposed (Goldenberg et al., 1959). Although this theory of an exclusive microvascular disease is widely debated, historical evidence for structural and functional microcirculation and related disturbances exist (Boulton et al., 2006). Also, studies have shown that capillary pressure is increased in the foot of people with diabetes due to arteriovenous shunting caused by sympathetic denervation (Deanfield et al., 1980; Flynn and Tooke, 1992; Boulton, 2000; Korzon-Burakowska & Edmonds, 2006). Collectively, these studies outline a critical role for microcirculation in ulceration.

With respect to diabetic foot ulcers, it is proposed that the impaired microcirculatory response may induce microcirculatory failure, resulting in tissue necrosis and ulceration (Flynn and Tooke, 1992; Korzon-Burakowska & Edmonds, 2006). Although microvascular disease may not be the single cause of pathogenesis of diabetic foot ulcers, the co-existence of abnormal microcirculatory function with both peripheral arterial disease and neuropathy may be associated with tissue damage (Flynn and Tooke, 1990; Boulton et al., 2006). This is supported by the evidence from studies that demonstrate the role of microcirculation in the development of ulceration, gangrene, necrosis and wound healing (Flynn and Tooke, 1992; Boulton et al., 2006; Levin et al., 2008; Lanting et al., 2017). Therefore, understanding functional abnormalities is of importance when studying diabetic foot ulcers.

## Injury, Inflammation and Soft-Tissues

To gain a better understanding of microcirculatory function and recognise appropriate methods to evaluate it, it is important to look at the bigger picture of the body’s defence, injury, inflammation and repair mechanisms. In the host defence mechanism, both lymphatic and blood vessels play an important role in an inflammatory response (Granger and Rodrigues, 2016; Parnham, 2016). Changes in the inflammatory mediators are known to correlate with the risk of developing a diabetic foot ulcer and inflammation is one of the earliest signs of ulcer (Lanys et al., 2021). Inflammation is a microcirculation-dependent tissue response to extrinsic and intrinsic stimuli (Granger and Rodrigues, 2016). During such an inflammatory response, the cardinal signs of inflammation that can be observed are heat (calor), pain (dolor), redness (rubor), and swelling (tumor), which may eventually lead to the loss of tissue function. In general, microcirculation is highly reactive to inflammatory response and plays a pivotal role in it as all components of the microvasculature such as the arterioles, capillaries, and venules respond and work towards the delivery of inflammatory cells to the injured or infected tissue/site (Granger and Senchenkova, 2010). The microvasculature isolates the infected or injured region from the healthy tissue and the systemic circulation, to facilitate tissue repair and regeneration (Johnson, 1973; Granger and Senchenkova, 2010; Bentov and Reed, 2014). The inflammatory responses of microcirculation include impaired vasomotor function, reduced capillary perfusion, leukocytes and platelets adhesion, activation of the coagulation cascade, enhanced thrombosis, increased vascular permeability, and an increased proliferation rate of blood and lymphatic vessels (Granger and Senchenkova, 2010). Other common microcirculatory changes result in shunting and hypoxia (reduced oxygen capacity of the tissues) caused by endothelial cell injury induced by a severe form of infection like sepsis, stasis of red blood cells due to vascular resistance, increased distances in oxygen diffusion in case of oedema owing to capillary leak syndrome (Guven et al., 2020).

In the foot, defence mechanisms (stimulation–response) plays a vital role. The role of microcirculation in wound repair and healing is well-realised (Shapiro and Nouvong, 2011; Ambrózy et al., 2013). Evidence suggests that despite the reasons behind an ulcer incident, the microcirculatory role in the process of healing remains the same and that the subpapillary perfusion plays a major role in the formation of granulation tissue, which was studied through the use of Laser Doppler Flowmetry system in patients with venous ulcers (Ambrózy et al., 2013). Microvasculature aids with tissue perfusion, fluid homeostasis, cutaneous oxygen delivery and recruiting collateral vessels to facilitate healing process (Bentov and Reed, 2014). Transcutaneous Oxygen Pressure (TcPo2) technique allows the measurement of cutaneous oxygen supply, which is found to be reduced in type 2 diabetic patients with the foot at risk of ulceration (Zimny et al., 2001). This was related to an impaired neurogenic blood flow regulation, which may contribute to capillary hypertension, endothelial dysfunction leading to oedema and skin damage (Zimny et al., 2001). Other non-invasive methods such as the measurement of skin perfusion pressure allow to assess healing (wound is likely to heal if pressure is above 30 mmHg) and to determine amputation levels (Sarin et al., 1991; Shapiro and Nouvong, 2011). Newer technology such as Laser Speckle Perfusion Imaging allows visualising the blood in the microvaculatore in and around the ulcer area, which may indicate the ability to heal (Shapiro and Nouvong, 2011). However, this device images cutaneous circulation to a depth no greater than 1 mm (Shapiro and Nouvong, 2011). While recent research focuses on assessing microcirculation to predict ulcer outcomes, further studies are needed to gain a deeper understanding of the microcirculatory changes in the ulcers with respect to the stages of healing for better prediction of wound healing.

Although the responses of the inflammatory system are regarded as defence mechanisms (stimulation–response) it may also be considered as a homeostatic system that operates continually to maintain organ and organism function (Tracy, 2006). Based on the dual nature of inflammation, stimulation–response and homeostatic, research suggest the use of biomarkers such as C-reactive protein or interleukin-6 to assess the activity level of the inflammatory process (Tracy, 2006). These biomarkers may represent normal homeostatic function, a response to a pathological condition or to both, which can take place to varying degrees depending on the differences in the person, time and condition (Tracy, 2006). Whilst in younger, healthier people, the biomarkers may likely represent the ongoing homeostatic activity, with increasing age and in the presence of underlying pathology such as chronic inflammatory changes due to diabetes or triggered atherosclerotic changes in cardiovascular conditions, these biomarkers may indicate a stimulation–response type inflammation (Payne, 2006; Tracy, 2006; Pahwa et al., 2020). Overall, there is consensus that inflammation biomarkers are independent predictors of the future occurrence of chronic disease outcomes and events (Tracy, 2006). Similarly, physiological markers such as skin temperature, galvanic skin response and perfusion measurements that indicate homeostatic and stimulation-response in relation to microcirculation may be pertinent to predict the future occurrences of chronic disease outcomes or events such as ulcers.

## Assessment of Microcirculation

Diabetic foot ulcers are multifactorial and there are new and emerging technologies that enable the assessment of these factors to aid prevention and management. Some of the methods are various nerve function tests (quantitative sensory testing, vibration perception, galvanic skin response and sudomotor activity testing), temperature measurement (infrared thermography), biomechanical properties measurements (plantar pressure and ultrasound indentation tests/elastography), macrovascular assessments (ankle-brachial index and toe brachial index) and microvascular assessments (TCPO2, laser doppler flowmetry, hyperspectral imaging and laser speckle contrast imager) and such (Pham et al., 2000; Naemi et al., 2017; Balasubramanian et al., 2020; Lung et al., 2020). However, in this review the main focus would be to discuss the assessment of microcirculation in tissue injury and inflammation to better understand its role in ulceration.

In the past, the key signs of inflammation were predominantly detected through mere observation. However, nowadays contactless and pain-free non-invasive techniques have facilitated objective assessment of inflammatory signs, tissue injury responses, repairs and healing. Laser Doppler flowmetry (LDF) technique is one such non-invasive technique, which allows assessment of microvascular blood flow when reflection and scattering of the laser light occurs due to the movement of the red blood cells (Nakamoto et al., 2012; Balasubramanian et al., 2020). Although the depth the laser penetrates is relatively low (∼1 mm), it is a useful device for the evaluation of cutaneous microcirculation. This device is gaining popularity in the field of research in diabetes, cerebrovascular conditions, Raynaud’s phenomenon and others. The use of LDF is being explored in dentistry as well, especially for perioperative procedures to gain a better understanding of soft tissue diagnosis. Apart from LDF, other non-invasive methods used to evaluate microcirculation are Laser Speckle Contrast Imager (LSCI) and photo-plethysmography. At times, since small fibre nerve functions and thermal changes influence microcirculation, methods such as quantitative sensory testing, skin electrodermal activity assessment and thermography are also used in conjunction with microvascular testing.

## Scope of This Review

This narrative review of literature focuses on key microcirculatory responses in relation to diabetic foot in order to understand some of the functional aspects of microcirculation. Firstly, search terms such as “Post-Occlusive Reactive Hyperaemia”, “PORH” “pressure-induced vasodilation”, “PIV” and “skin blood flow” “local application of pressure”, “LDI flare” and “axon-mediated flare” were listed and used to identify articles (the search was not limited to these terms only). PubMed and Medline databases were searched to identify relevant publications in journals. Secondly, the reference lists of the selected articles were scrutinised to find additional studies. However, the data sources were not limited to articles published in journals, but also included grey literature. The sources for grey literature included: 1) Reports from International Diabetes Federation and Diabete UK 2) Websites of equipment manufacturers (Perimed AB, Moor Instruments, FLIR and Impeto Medical Solutions) 3) OpenGrey, and 4) Google. The articles of interest from MEDLINE, PubMed, and PubMed Central (PMC) included in the review were identified through the initial phase of title and abstract sifting. Subsequently, after the title and abstract sifting, relevant articles that adequately described cutaneous microcirculatory responses were retrieved for further study. Later, the data were extracted from relevant articles. Specific insights generated from the literature are presented and discussed below.

## Various Microcirculatory Responses and Their Association in Diabetes Foot-Related Complications

The foot is continuously under mechanical stress due to weight-bearing activities of daily living such as walking, exercise, and standing. It is exposed to various trauma, physical injury due to sudden or violent action, exposure to dangerous toxins or repetitive mechanical stress. Some of the extrinsic factors for trauma are thermal (Example: hot surfaces), mechanical (Example: repetitive damage from ill-fitted shoes), and chemical (Example: corn treatments) (Boulton, 2000; Armstrong and Lavery, 2005; Boulton et al., 2006; Vanderah, 2007; Hawke and Burns, 2009). On the other hand, some of the intrinsic factors that contribute to the risk of trauma are foot deformity and glycation related changes in case of diabetes.

Both neuro and vascular aspects are essential for healthy foot function. The nerves of the feet can respond to the thermal, mechanical and chemical stimuli, provoking a reflex withdrawal from the respective harmful stimulus (Hawke and Burns, 2009). For instance, jerking the foot away from a sharp object. This protective mechanism may be absent due to neuropathy in people with diabetes (Boulton et al., 2006; Hawke and Burns, 2009). On the other hand, microcirculation is important for tissue injury response to stimuli such as local heat or pressure (Abraham et al., 2001; Korzon-Burakowska & Edmonds, 2006). Such neurovascular mechanisms of the foot appear to play a vital role to prevent tissue injuries.

Previous research shows that there are certain protective microcirculatory responses to stimuli, which are controlled by neural mechanisms, metabolic aspects, hormones and chemicals (Guyton, 1991). A microcirculatory hyperaemic response is induced on the application of a stimulus. This transient hyperaemic response to various stimuli, witnessed by an increase in blood perfusion is one of the measures to assess microcirculatory function known as reactive hyperaemia. Reactive hyperaemia is an indicator of the intrinsic ability of an organ or tissue to locally autoregulate its blood supply, which is found to be impaired in people with diabetes (Flynn and Tooke, 1992; Korzon-Burakowska & Edmonds, 2006; Merrill, 2008; Klabunde, 2012). For the purpose of this review, based on the select stimuli, the microcirculatory responses observed are stratified into:

1) Vasodilation in response to occlusive ischemia or Post-Occlusive Reactive Hyperaemia (PORH)
2) Microcirculatory response to locally applied pressure;
  (a) Pressure-induced vasodilation (PIV);
  (b) Reduced skin blood flow;
3) Interplay between microcirculation and temperature -vasodilation in response to local heating

The reviewed studies demonstrate the inability of cutaneous microcirculation to respond normally to non-painful stimulation, such as the application of pneumatic pressure, local pressure and local heating in people with diabetes (Fromy et al., 2002). This may be significant in understanding tissue response to injuries. During incidents of prolonged pressure, injury or infection, more demands are made upon the capillary circulation (Flynn and Tooke, 1992; Abraham et al., 2001). Owing to the microcirculatory dysfunction, the hyperaemic response may be impaired and tissue demands are not met (Flynn and Tooke, 1992). Vascular insufficiency to the tissues that leads to breakdown may contribute to adverse complications and increase the risk of ulceration (Flynn and Tooke, 1992). Nevertheless, there are very limited studies that evaluate the vasodilatory responses to stimuli in subjects with diabetes. Furthermore, only a handful number of research articles address these vasodilatory responses in diabetic foot syndrome, including ulcerated and non-ulcerated cohorts. Key articles on this subject were appraised and discussed in this review.

### Vasodilation in Response to Occlusion or Post Occlusive Reactive Hyperaemia (PORH)

Reactive hyperaemia to occlusion is the transient increase in blood flow in the organ or tissue that occurs following a brief period of arterial occlusion. During the process of occlusion, the blood flow goes to a biological zero that is defined as the “no flow” Laser Doppler signal during a PORH test. Following the release of the occlusion, blood flow rapidly increases, which is reactive hyperaemia (Klabunde, 2012). This process is known as post-occlusive reactive hyperaemia (PORH). During the hyperaemia, the tissue becomes re-oxygenated and reperfusion occurs. Simultaneously, the vasodilator metabolites are removed from the tissue, which restores the vascular tone of the resistant vessels causing the blood flow to return to normal (Klabunde, 2012). The longer the period of occlusion, the greater the metabolic stimulus for vasodilation leading to an increase in peak reactive hyperaemia and duration of hyperaemia (Guyton, 1991; Larkin and Williams, 1993; Klabunde, 2012). Based on the time taken to occlude the blood supply to the tissue from few seconds to several hours, the blood flow post-occlusion increases four to seven times in the tissue than normal and lasts from few seconds to hours in relation to the initial occlusion time (Guyton, 1991). Additionally, depending upon the organ or tissue, maximal vasodilation as indicated by peak flow varies (Klabunde, 2012).

PORH is predominantly an endothelial-dependent process, however, it also aids combined assessment of both endothelial-dependent and independent function (Maniewski et al., 2014; Lanting et al., 2017). Hyperaemia occurs because of the shear stress, the tangential frictional force-acting at the endothelial cell surface caused by arterial occlusion (Maniewski et al., 2014). A mechanical stimulation occurs when the shear stress vector is directed perpendicular to the long axis of the arterial vessel (Maniewski et al., 2014). The endothelium responds to this mechanical stimuli, thereby, releasing vasodilatory substances (Maniewski et al., 2014). The factors that are known to contribute to vasodilation are myogenic, neurogenic, and other local factors, such as potassium ions, hydrogen ions, carbon dioxide, catecholamines, prostaglandins, and adenosine (Maniewski et al., 2014; Lanting et al., 2017). Few studies mention that endothelial nitric oxide and other endothelium-derived agents, such as prostaglandins and endothelium-derived hyperpolarizing factors are known to be involved in the mechanism of PORH (Maniewski et al., 2014; Carasca et al., 2017; Marche et al., 2017). However, some researchers contend that nitric oxide and prostaglandins may not be contributing to the mechanism (Cracowski et al., 2011; Maniewski et al., 2014). It is argued that whilst nitric oxide is known to play a major role in the vasodilation of macrovessels, endothelium-derived hyperpolarizing factors are found to play a substantial role in the dilation of microvessels (Quyyumi and Ozkor, 2006; Cracowski et al., 2011). Apart from these substances, the sensory nerves make a vital contribution to the PORH mechanism (Larkin and Williams, 1993; Lorenzo and Minson, 2007; Cracowski et al., 2011; Lanting et al., 2017; Marche et al., 2017). To summarise, various studies have shown that PORH response is elicited with temporary tissue hypoxia upon occlusion through the accumulation of vasodilators (substances that cause the blood vessels to dilate or expand) and other complex factors that are myogenic, endothelial, neurogenic and metabolic (Guyton, 1991; Klabunde, 2012; Lanting et al., 2017).

The PORH test has a wide range of applications. Previously, PORH has been used to assess microcirculatory function in people with arterial diseases, certain ophthalmologic conditions and cardiovascular disorders (Morales et al., 2005; Maniewski et al., 2014; Carasca et al., 2017). It is impaired in people with peripheral arterial disease and has been associated with increased cardiovascular risk (Morales et al., 2005). The test was observed to be useful as an early marker of cardiovascular damage (Busila et al., 2015). PORH test is also used to assess the altered microvascular reactivity in patients with advanced renal dysfunction (Busila et al., 2015). Besides, the use of the PORH test has also been explored in the area of diabetes.

A limited amount of research has been conducted in people with diabetes using PORH measures, both in type 1 and 2. PORH vasodilation is significantly decreased in patients with type 1 diabetes (Marche et al., 2017). In 1986, Rayman et al. demonstrated the impaired hyperaemic response to injury in people with diabetes for the first time (Rayman et al., 1986). Prolongation of the hyperaemic reaction and decrease in response was observed in patients with insulin-dependent diabetes and peripheral occlusive arterial disease (Maniewski et al., 2014). PORH is known to be impaired not only in adults but also in children with type 1 diabetes (Schlager et al., 2012). The results from children in terms of diabetic foot complications is as important as the studies conducted in adults because of two main reasons. Firstly, although this segment of the population is less likely to be vulnerable to foot complications at a younger age, but they are likely to develop complications as they advance in age. Therefore, understanding the microvascular reactivity from an earlier period may prove to be useful. Secondly, this particular study explored other less commonly assessed variables such as biological zero and reperfusion time, which can shed more light on understanding PORH. It was identified that peak perfusion was higher and biological zero was lower in children with type 1 diabetes in comparison to the controls. A key implication from this study was that higher peak perfusion might reflect a decline in the vasoconstrictive ability of arteriolar smooth muscle cells upstream of capillary beds in children with type 1 diabetes (Schlager et al., 2012).

Few studies have explored PORH more specifically in diabetic foot complications (Cheng et al., 2004; Barwick et al., 2016; Lanting et al., 2017). The presence of peripheral sensory neuropathy in people with type 2 diabetes is found to be associated with altered PORH in the foot (Barwick et al., 2016). A study on the relationship between active or previous foot complication and PORH measured by LDF in people with type 2 diabetes revealed that the increase in time to Peak, which is a variable that shows the time taken for a maximum flux post occlusion, increased the likelihood of a participant having a history of foot complication by 2% (Lanting et al., 2017). This association was not reflected in people with an active foot ulcer (Lanting et al., 2017). These findings in a cohort with type 2 diabetes with a previous history or existing foot-related complications support the need for further investigation into the relationship between measures of microvascular function and development of diabetic foot complications, prospectively (Lanting et al., 2017). Considering this evidence, it seems that PORH is an interesting microcirculatory mechanism that may be useful to assess a foot at risk. In future, their application may be a useful indicator for determining the future risk of diabetic foot complications, especially with ulcer prediction and prevention of amputation.

### Microcirculation in Response to Local Application of Pressure

In the foot, the areas prone to high pressure such as the heel, the great toe and areas under the metatarsal heads are at risk of ulceration (Veves et al., 1992; Ledoux et al., 2013). Based on this, many weight-bearing activities were considered to be a contraindication to people with neuropathy (Kluding et al., 2017). However, this has recently changed as there is emerging evidence of positive adaptations of the musculoskeletal and integumentry system to overload stress (Kluding et al., 2017). Literature suggests that peripheral neuropathy may no longer be a hindrance to promoting weight-bearing activity as it did not lead to significant increases in foot ulcers (LeMaster et al., 2008). However, in people with diabetes various other factors may interplay with pressure such as increased stiffness of tissues, aging related changes, presence of other comorbidities, mobility and vascular issues. Studies show that the accumulated mechanical stimulus affected blood perfusion in the foot and should be considered when assessing the risk of developing ulcers (Ledoux et al., 2013; Pu et al., 2018). However, more understanding on the relationship between pressure stimulus and microvascular responses could shed more light on the effect of different levels of accumulated mechanical stimulus on microvascular response and their significance in an ulcer incident.

Responses to local mechanical stresses are mediated through a considerable number and variety of cutaneous receptors and some of these receptors are connected to the small fibres (Abraham et al., 2001). The vasodilation to pressure strains not only occur for noxious stimuli but also non-noxious stimuli applied over a period (Abraham et al., 2001). Local pressure strain to the skin is recognised to play a vital role in cutaneous microcirculatory impairment (Fromy et al., 2000; Abraham et al., 2001). It is presumed that this may be linked to the development of cutaneous lesions such as pressure sores and diabetic foot ulcers (Abraham et al., 2001; Fromy et al., 2002). Two important microcirculatory responses to locally applied pressure identified through the literature review are discussed below.

#### Pressure-induced Vasodilation

The transient increase in cutaneous blood flow initially before it decreases in response to a progressive locally applied pressure strain is known as pressure-induced vasodilation (PIV). This microcirculatory response appears to be a protective cutaneous response that relies on the excitation of unmyelinated afferent nerve fibres (Fromy et al., 2002; Koïtka et al., 2004; Körei et al., 2016). PIV is considered to be more than a transient phenomenon and an important physiological response allowing the skin to respond adequately to mechanical stimuli (Abraham et al., 2001). Cutaneous receptors in the skin respond to local mechanical stresses such as local pressure strain and these receptors are found to be of mechanothermal nature (Fromy et al., 2002). This response is noted to be compromised in the aging population (Fromy et al., 2010; Fouchard et al., 2019). Furthermore, the impairment of PIV is postulated to contribute to the development of lesions such as pressure ulcers and diabetic foot ulcers (Abraham et al., 2001; Saumet, 2005; Vouillarmet et al., 2019).

The interplay between biomechanical factors and physiological responses is well-realised in the development of pressure ulcers, including in people with diabetes. Current studies highlight PIV in relation to the development of pressure ulcers or decubitus ulcers in the sacral region. As discussed above, one of the key implications from the studies on PIV is that it is a protective mechanism without which certain pressure-associated lesions may develop and plausibly this could explain the high risk of decubitus and plantar ulcers in people with diabetes (Abraham et al., 2001; Fromy et al., 2002; Bergstrand, 2014). Although pressure ulcers and plantar ulcers may differ in many ways, one of the key causal pathways to foot ulceration is somatic motor neuropathy that leads to small muscle wasting, foot deformities, loss of sensation, increased plantar pressure and repetitive trauma resulting in neuropathic foot ulcer (Armstrong and Lavery, 2005). This suggests that local pressure strain increases the vulnerability of the foot to ulcerate. Similar to pressure ulcer development, reduced physiological responses may induce local ischaemia and reperfusion injury in the foot (Flynn and Tooke, 1992; Coleman et al., 2014). A similar role of reduced microcirculatory responses in foot ulcer development is widely discussed in the literature (Flynn and Tooke, 1992; Boulton et al., 2006; Korzon-Burakowska & Edmonds, 2006). This knowledge can potentially be translated to diabetic foot ulcer prediction to see if the microcirculatory response to local pressure and plantar pressure have any association. This also accords with other observations, which showed that people with impaired or absent PIV are known to be at a higher risk to develop pressure ulcers (Fromy et al., 2002; Braden and Blanchard, 2007; Bergstrand, 2014). Evidence shows that decreased hyperaemic response and absence of PIV is known to increase the risk of pressure ulcers (Bergstrand et al., 2014). However, very limited research is available on PIV in human hand and feet in relation to diabetes (Abraham et al., 2001; Koïtka et al., 2004).

A particular study by Koitka et al. (2004) observed PIV at the foot level in people type 1 diabetes (Koïtka et al., 2004). Since low skin temperature in people with diabetes is known to interfere with microcirculation, this research was performed in warm conditions of 29.5± 0.2°C (Koïtka et al., 2004). The cutaneous blood flow was studied at warm conditions using laser Doppler flowmetry on the first metatarsal head in response to applied pressure at 5.0 mmHg/min and PIV was found to be absent at foot level in people with type 1 diabetes whereas it existed in healthy subjects at 29.5±0.2°C (Koïtka et al., 2004). These findings were attributed to an interaction between functional changes in C-fibres and the endothelium in people with diabetes (Koïtka et al., 2004). A similar study found PIV to be absent at low skin temperature even in healthy subjects (28.7±0.4°C) (Fromy et al., 2002). It was explained that a skin temperature close to 34°C was optimal for the evaluation of skin vasomotor reflexes in the lower limb and the nervous receptors involved in the PIV development are mechanothermal, and not only mechanical (Fromy et al., 2002). The results from Koitka et al. (2004) revealed that in the same subjects the non-endothelial-mediated response to sodium nitroprusside was preserved, whereas the endothelial-mediated response to acetylcholine was impaired (Koïtka et al., 2004). Therefore, suggesting the relevance of endothelial dysfunction to PIV. Also, a previous study on PIV found that the absence of vasodilatory axon reflex response to local pressure strain when the capsaicin-sensitive nerve terminals were pre-treated with local anaesthetic or chronically applied capsaicin (Fromy et al., 1998). The capsaicin-sensitive nerve fibres are the small nerve fibres and their role in neuropathic pain and related complications, especially in people with diabetes is well-established (Boulton et al., 2006). Thus, the researchers speculated that the PIV, which is associated with the stimulation of small fibre nerves, could be a missing link between neuropathy and foot ulcers in diabetes (Koïtka et al., 2004). In support of this, several studies have demonstrated that damage to C-fibres have a great impact on skin, with disrupted blood flow predisposing to foot ulcers (Vinik et al., 2001; Caselli et al., 2003; Boulton et al., 2006; Themistocleous et al., 2014). As previously discussed, impaired microcirculatory response to local pressure strain may potentially make people with diabetes more vulnerable to pressure strains and explain the high prevalence of foot ulcer that occurs in diabetic patients (Koïtka et al., 2004).

The insights from the above-discussed studies suggest that PIV is absent at the foot level in people with diabetes. Identifying the point or stage of the disappearance of PIV in the foot, during the disease progression through prospective studies, may help in understanding the progression of neurovascular dysfunction in the foot. On the other hand, since PIV may be absent from an earlier stage, its capability to indicate risk for ulceration is disputable and needs further research. Also, the current study has observed PIV only at two sites, which was the head of the first metatarsus and the area over the internal ankle bone in a small sample size. More research is required to explore various regions of the plantar aspect of the foot, especially in areas subject to increased plantar pressure. The findings from such research can aid to comprehend the association between PIV and plantar ulcers and help identify foot at risk. Furthermore, it may aid to bridge the research gap to understand the role of microcirculation in the development of diabetic foot ulcers.

#### Reduced Skin Blood Flow to Locally Applied Pressure

As discussed earlier, PIV allows the skin blood flow to increase in response to locally applied pressure. In the absence of the transient PIV response, the cutaneous blood flow is observed to progressively decrease with the application of increasing local pressure (Fromy et al., 2002). The observed cutaneous blood flow in response to locally applied pressure is found to be impaired in people with diabetes owing to the combined effects of low cutaneous temperature and alterations in microcirculatory function (Fromy et al., 2002). Additionally, the presence of neuropathy may aggravate the condition (Fromy et al., 2002). This study used a laser Doppler flowmetry system and applied local pressure using a specially designed apparatus at the internal anklebone allowing for a 5.0 mmHg/min rate of pressure increase (Fromy et al., 2000; Fromy et al., 2002). The skin blood flow decreased significantly from baseline at much lower applied pressure of 7.5 mmHg in people with diabetes in groups without neuropathy and with subclinical or clinical neuropathy at 6.3 mmHg in comparison to the healthy controls at 48.8 mmHg (Fromy et al., 2002). The large difference between these pressures reported within this study indicate a plausible association between decreased skin blood flow to local pressure and the development of decubitus and plantar ulcers (Fromy et al., 2002). This hypothesis is consistent with the one proposed by Koitka et al. (2004) who suggested an association between microcirculatory dysfunction and the high prevalence of foot ulcer (Koïtka et al., 2004). They also postulate that the arterial wall and surrounding tissues are very compressible in people with diabetes making them vulnerable to the development of pressure ulcers (Fromy et al., 2002; Coleman et al., 2014). The application of this knowledge to understand the role of microcirculation in foot ulceration may potentially be useful.

Although the collated findings reveal the possibility of decreased skin blood flow and PIV to be associated with pressure ulcer development, more research is needed to understand the mechanism in relation to diabetic foot complications. The aetiology for decubitus ulcer and plantar ulcers may vary, nevertheless, pressure remains as a common contributing factor in both the incidents. Studies suggest pressure-induced local ischaemia and reperfusion injuries in relation to both pressure ulcers and diabetic foot ulcers (Flynn and Tooke, 1992; Korzon-Burakowska & Edmonds, 2006; Coleman et al., 2014; Shahwan, 2015). Understanding PIV, reduced skin flow and other microcirculatory responses in various regions prone to diabetic foot ulcers and in relation to plantar pressure during standing or walking are important. The need for such a study is further supported by the evidence from a study that identified subjects who lacked PIV and reactive hyperaemia in response to locally applied pressure, to be particularly vulnerable to pressure exposure (Bergstrand, 2014; Bergstrand et al., 2014). These subjects were stratified to be at a higher risk for pressure ulcer development (Bergstrand, 2014; Bergstrand et al., 2014). Thereby, translating the knowledge generated from the studies on microcirculatory responses in the development of pressure ulcers to diabetic foot ulcers can prove to be useful.

### Interplay Between Microcirculation and Temperature - Vasodilation in Response to Local Heating

While specific literature on the microcirculatory responses and temperature changes in response to plantar skin tissue injuries and healing are limited, previous studies reviewed microcirculatory assessments in various organs in people with diabetes. The knowledge of microcirculatory responses to temperature changes in other organs, can reveal that external stimuli causes an increased microvascular demand. This showcases the role of cutaneous microcirculatory response in tissue injuries and healing.

When injuries and repair occur, monitoring the conditions between the skin, soft tissues or even after skin grafts can aid better prognosis. A study explored the proposed theory that conducive interface conditions between soft tissue and prostheses are necessary for a better outcome with prosthodontic treatment. This study by Nakamoto et al. (2012) focused on the gingiva and mucosa surrounding anterior implants and both LDF and thermographs were concurrently used to elucidate the relationship between temperature and blood flow as peri-implant soft tissues are often portrayed to have decreased blood flow because of the lack of blood supply from the periodontal ligament. The study also analysed the morphological changes of the cutaneous microvasculature and temperature changes between participants with and without bone grafting associated with implant placement. The findings suggested that soft tissue around implants showed decreased blood flow compared with periodontal tissue in adjacent natural teeth, despite the absence of clinical signs such as chronic inflammation. The study also highlighted the significance of bone quality to maintain blood flow in the soft as the area around implants with bone grafting showed significantly reduced blood flow. Many research studies suggest that microcirculatory blood flow is influenced by thermal changes and reportedly increases in proportion to temperature to an extent, which is not limited to dentistry but also in studies on other cutaneous microcirculation (Molnár et al., 2015). However, the observed results by Nakamoto et al. (2012) were contrary to this popular idea. The suggested explanation for this was the involvement of deeper structures that modified the thermal properties and the usually observed increase in temperature was often associated with inflammation due to infection such as periodontitis but not in case of tissue surrounding implants (Baab et al., 1990).

Although the skin and the oral mucosa have certain similarities and differences anatomically, they have some comparable physiological properties. For instance, they play a crucial role in the prevention of infections and act as a barrier against exogenous or endogenous substances, pathogens, and mechanical stresses (Liu et al., 2010). The dysfunction of these barriers can compromise the integrity of the underlying tissue as well. The combination of findings from the study provides some support for the conceptual premise that the simultaneous measurements of blood flow and temperature are useful to evaluate the microcirculation of soft tissue behaviour in injury and healing, and its significane even in the absence of noticeable signs chronic inflammation. A similar study compared the peripheral blood flow in the lower limbs during the local heating tests with different temperature protocols in people with diabetes mellitus and healthy participants (Filina et al., 2017). The LDF was used to evaluate the adaptive changes of the microvascular bed during thermal tests and the detection of the preclinical stage of trophic disorders owing to disruption in nutritional or nerve supply (Filina et al., 2017). Research suggest that in the feet of patients with diabetic neuropathy, total skin blood flow is increased due to an increased shunt flow due to denervation (Harpuder et al., 1940; Schaper et al., 2008). Further study in the area has shown that the increased anastomotic shunt flow lead to either under- or over perfused nutritive capillaries (Netten et al., 1996). Skin temperature measurements and LDF were performed to record mainly shunt flow and capillaroscopy to study nailfold capillary blood flow (Netten et al., 1996). The study showed that in insulin-dependent diabetic patients with neuropathy, the baseline skin temperature and capillary blood-cell velocity was higher in comparison to those without neuropathy and healthy control subjects (Netten et al., 1996). The findings from the study highlighted the presence of hyperperfused nutritive capillary circulation in the feet of patients with diabetic neuropathy favouring the previously discussed hyperdynamic hypothesis and in contradiction to the capillary steal phenomenon to explain the decreased healing potential in diabetic neuropathic foot ulceration.

As suggested by previous research, microcirculatory and temperature measurements might become useful techniques to evaluate healthy, infected, injured, inflamed and treated skin and soft tissues of the foot (Netten et al., 1996; Gatt et al., 2018; Gatt et al., 2020). But, there is abundant room for further progress in determining if these two measurements may be useful for the diagnosis or prognosis of foot ulcers. Such research may aid to draw a margin between the compromised tissue and the surrounding healthy tissue when determining the course of treatment, surgery or even amputation. Furthermore, comparative studies conducted on healthy vs inflamed/injured tissue in the foot can help to identify early signs of dysfunction, inflammation and injury in a foot in order to effectively manage the condition. For instance, Ren et al. (2021) explored the stimulation of microcirculation using simple thermal stimuli such as infrared and warm bath in healthy adults to explore the options in hope to design interventions to promote better circulation in the lower extremities of the body in the geriatric population and those suffering from diabetes who are likely to have impaired microcirculation (Ren et al., 2021).

The vasodilation in response to local heating and the neurogenic flare response to nociceptive stimuli is mediated by an axon reflex involving C-fibres. This is studied using the laser Doppler imager (LDI) and the induced flare response is known as the LDI flare. The LDI flare area which is the area with the hyperaemic response is known to be reflective of the small fibre function. Therefore, the size of the LDI flare is known to be dependent on the C-fibre function and the underlying skin small fibre neural network and its extent (Green et al., 2010; Vas et al., 2012). Whereas, the LDI max (perfusion) in the skin immediately beneath the heating probe is shown to be mediated by non-neurogenic means and to reflect the endothelial function (Green et al., 2010; Vas et al., 2012). Therefore, the intensity of the hyperaemic response depends on the microvascular ability to vasodilate. The site commonly studied is the dorsum of the feet because the underlying skin is less influenced by the thermoregulatory blood flow due to the absence of arteriovenous anastomoses (Braverman, 2000). The method used to assess this reflex involves local skin heating to 44°C for 20 min or 6 min in a stepwise fashion: 44°C for 2 min, 46°C for 1 min and finally 47°C for 3 min in a temperature-controlled room to evoke the flare followed by scanning the site using an LDI to measure the area (Krishnan and Rayman, 2004; Green et al., 2010; Vas et al., 2012). Another technique is also known to be used to observe the hyperaemic response to local heating. This involves the use of a skin-heating probe filled with deionized water and heating to 44 °C to assess heat-induced vasodilation. In summary, the LDI flare test in subjects shows reduced microcirculatory response as well as a neurogenic flare in people with either type 1 or two diabetes (Krishnan and Rayman, 2004; Vas et al., 2012). It facilitates early diagnosis of C-fibre dysfunction even before its detection by other available methods such as the quantitative sensory testing, which focuses on the testing of sensory abnormalities in the areas of temperature change sensation, vibration, and pain threshold testing (Example: Using equipment named Computer Aided Sensory Evaluator–IV - case IV) (Krishnan and Rayman, 2004). Therefore, the heat provocation or LDI flare test is commonly used with a focus on LDI flare for the assessment of C-fibre function than with a concentration on the LDI max for evaluating the microcirculatory function. However, the test can be used to assess not only C-fibre function but also microcirculation, and additionally investigate their association in neuropathy (Vas et al., 2012; Marche et al., 2017). This can further clarify the link between microcirculation impairment and tissue damage in light of impaired sensation.

## Conclusion

Microcirculation plays a vital role in homeostatic and defence states during tissue injury and inflammation. Firstly, the most obvious finding to emerge from this review is the protective role of microcirculation. Secondly, the impairment of microcirculation and the possibility of it being the missing link in the chain of events that leads to foot ulceration in people with diabetes is clearly supported by the current findings. Thirdly, assessment of microcirculatory structural damages might be complex, however, the insights emerged from this review has shown that there are responses such as post-occlusive reactive hyperaemia, pressure-induced vasodilation and vasodilation to local heating (LDI flare) that are simple to assess. In conclusion, a thorough understanding of the microcirculatory function and its impaired reactive mechanisms is imperative and will contribute extensively to understanding the soft tissue biomechanics and aid to devise strategies for comprehensive assessment of the diabetic foot. This, in turn, will aid in prevention and early diagnosis of ulcers, thereby, reducing amputations.

## Abbreviations

PORH, Post-Occlusive reactive hyperaemia; PIV, Pressure-Induced Vasodilation; LDI, Laser Doppler Imager; GDP, Gross Domestic Product.
